# Creating healthy food environments through global benchmarking of government nutrition policies and food industry practices

**DOI:** 10.1186/2049-3258-72-7

**Published:** 2014-03-05

**Authors:** Stefanie Vandevijvere, Boyd Swinburn

**Affiliations:** 1 Department of Epidemiology and Biostatistics, University of Auckland, School of Population Health, Auckland, New Zealand; 2 Scientific Institute of Public Health, Department of Public Health and Surveillance, Brussels, Belgium; 3 WHO Collaborating Centre for Obesity Prevention, Deakin University, Melbourne, Australia

## Abstract

Unhealthy processed food products are increasingly dominating over healthy foods, making food and nutrition environments unhealthier. Development and implementation of strong government healthy food policies is currently being circumvented in many countries by powerful food industry lobbying. In order to increase accountability of both governments and the private sector for their actions, and improve the healthiness of food environments, INFORMAS (the International Network for Food and Obesity/non-communicable diseases (NCDs) Research, Monitoring and Action Support) has recently been founded to systematically and comprehensively monitor food environments and policies in countries of varying size and income. This will enable INFORMAS to rank both governments and private sector companies globally according to their actions on food environments. Identification of those countries which have the healthiest food and nutrition policies and using them as international benchmarks against which national progress towards best practice can be assessed, should support reductions in global obesity and diet-related NCDs.

## Background

Every individual should have the right to get easily accessible, readily available, relatively inexpensive and sufficiently promoted healthy foods. However, in general, unhealthy processed food products are increasingly dominating over healthy foods, making food environments unhealthier, and are profoundly multiplying their effects on health [1,2]. Food environments, most broadly, include a combination of physical, economic, policy and socio-cultural surroundings, opportunities and conditions that influence food choices [3]. Implementation of strong government healthy food and nutrition policies is lagging in many countries, mainly due to clever and effective food industry influences. This was observed in Europe, where the front-of-pack traffic light labelling, to improve interpretation of nutrition information by consumers, was not implemented after huge lobbying campaigns by the food and drink industries [4]. Several Latin American governments recently also attempted to get healthy food policies developed and implemented, but most (apart from Mexico which very recently introduced a 1 peso per liter tax on soft drinks and a 8% tax on junk food) did not yet succeed due to strong industry pressure [5]. Despite this, it is encouraging that this detrimental food industry pressure was recently recognised as one of the biggest problems facing health promotion by Dr Margaret Chan in her opening speech at the 2013 Health Promotion Conference in Helsinki [6], and by a group of renowned scientists who recently published the Bellagio declaration on the undermining effect of food corporations on healthy food policies and a set of key actions for governments, the World Health Organisation (WHO), international agencies, researchers, foundations and civil society in order to address this problem [7].

## Discussion

In an ambitious effort to shift current diets in the direction of meeting dietary guidelines, INFORMAS (the International Network for Food and Obesity/non-communicable diseases (NCDs) Research, Monitoring and Action Support) [3] has recently been founded and aims to increase accountability of both governments and the food industry for their actions on food environments and to give a stronger voice to consumers [8] to advocate for healthy food environments and diets. INFORMAS aims to achieve this through monitoring and benchmarking key aspects of food environments, as well as policies, actions and practices of governments and private sector organisations impacting on those. The standardised and stepwise INFORMAS monitoring approaches have recently been published as a supplement in *Obesity Reviews*[9-17].

This new initiative aims for monitoring to be highly policy responsive, to occur at low cost and be sustainable, to make results available online and open access in different formats for different stakeholders and to be complementary to monitoring efforts of the WHO. The global political commitment made in May 2013 towards a comprehensive plan for the prevention and control of NCDs and for a monitoring framework to measure progress on 25 indicators towards 9 targets [18] is deficient in monitoring food environments and policies.

Systematic and comprehensive monitoring in countries of varying size and income should enable INFORMAS to rank both governments and private sector companies globally according to their actions on for example decreasing salt, sugar and fat levels in foods, restricting unhealthy food advertising targeted at children, providing clear and easily interpretative front-of-pack nutrition labels, improving the nutritional quality of foods provided and sold in different settings (especially schools) and increasing the relative availability and affordability of healthy versus unhealthy foods in communities. Best practice exemplars or benchmarks will be derived from this international monitoring and progress of countries, and companies, on improving food environments will be compared against those. To assess government policies and actions towards good practice, INFORMAS has proposed a government healthy food environment policy index (Food-EPI) [9]. This index, more than a tool for monitoring alone, aims to increase engagement with both policymakers, as well as the public health nutrition community in participating countries. Its impact on catalysing policy responses is expected to be significant and will be measured. A separate assessment of private sector actions and practices [10] draws on experience from the recently launched Access to Nutrition index (ATNI) [19], supplemented with the measurement of less visible practices, such as lobbying, political donations and corporate philanthropy. This assessment may give insight in the best strategies to overcome the power of the food industry currently circumventing the implementation of strong public health nutrition policies. This assessment may give insight in the best strategies to overcome the power of the food industry currently circumventing the implementation of strong public health nutrition policies.

## Conclusions

INFORMAS hopes to closely engage with different stakeholders (governments, private sector, researchers, NGOs, media, public), increase levels of accountability of governments and the food industry, and stimulate more effective policies and actions to improve the healthiness of food environments. In addition, rich international databases will allow a deeper understanding of how food policies and environments affect obesity and NCDs and allow evaluation of the impacts of new food policies and actions on food environments, obesity and NCD risk factors. This new monitoring initiative should help to identify the best strategies to improve access and availability of healthy diets at affordable prices for all individuals. In the longer run INFORMAS should identify equity and sustainability indicators as well, which could help to come to more integrated food policies meeting the challenges of chronic disease, climate change, loss of biodiversity, resource efficiency and food security.

## Competing interests

The authors declare that they have no competing interests.

## Authors’ contributions

SV drafted the manuscript. BS reviewed the draft. Both authors read and approved the final manuscript.
